# P-935. A Mobile-Based Platform for Real-Time Antimicrobial Surveillance, Prescribing Audit, and Stewardship Intervention Tracking in a Resource-Limited Indian Tertiary Care Hospital

**DOI:** 10.1093/ofid/ofaf695.1138

**Published:** 2026-01-11

**Authors:** Jose J Kochuparambil, Dona Ann Varkey

**Affiliations:** MQMH, Pala, Kerala, India; Mary Queens Mission Hospital, Anakkallu, Kerala, India

## Abstract

**Background:**

Optimizing antimicrobial use is essential to curb resistance and improve clinical outcomes. In resource-limited hospitals, stewardship programs often lack real-time data, structured audit tools, and feedback mechanisms. This study evaluated a mobile-based antimicrobial stewardship (AMS) platform for enhancing prescribing practices, intervention tracking, and response timeliness in a 250-bedded tertiary care hospital in South India.

**Figure:**
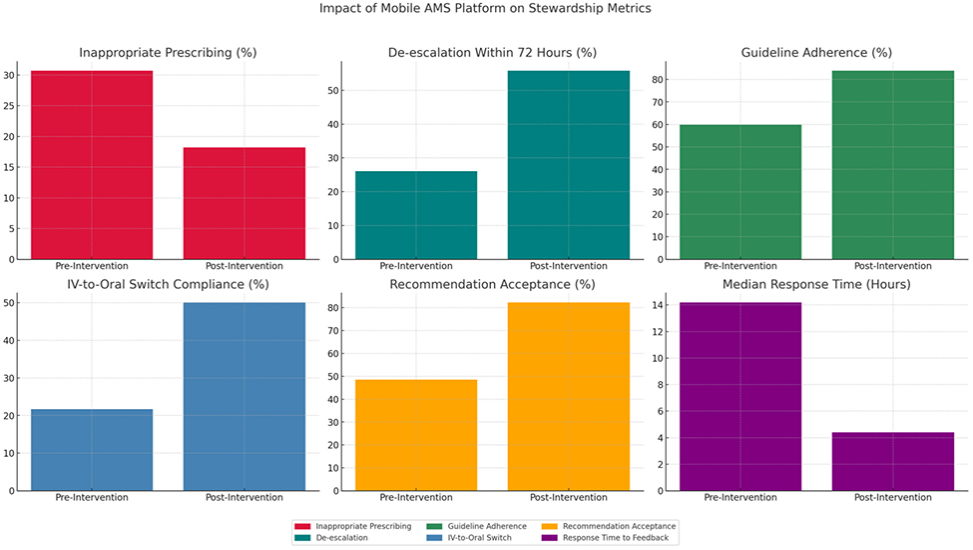
Impact of Mobile AMS Platform on Stewardship Metrics This graph illustrates the improvement in key antimicrobial stewardship indicators following the implementation of a mobile-based AMS platform in a tertiary care hospital in South India. Inappropriate prescribing rates declined from 30.7% to 18.2%, while de-escalation within 72 hours improved significantly from 26.1% to 55.8%. Guideline adherence increased from 59.8% to 83.9%, and IV-to-oral switch compliance rose from 21.7% to 50.1%. Acceptance of stewardship recommendations improved from 48.5% to 82.3%. Most notably, the median response time to AMS interventions was reduced from 14.2 hours to 4.4 hours. These results demonstrate the platform’s ability to enhance prescribing quality, clinical responsiveness, and interdisciplinary collaboration in a resource-limited setting.

**Figure:**
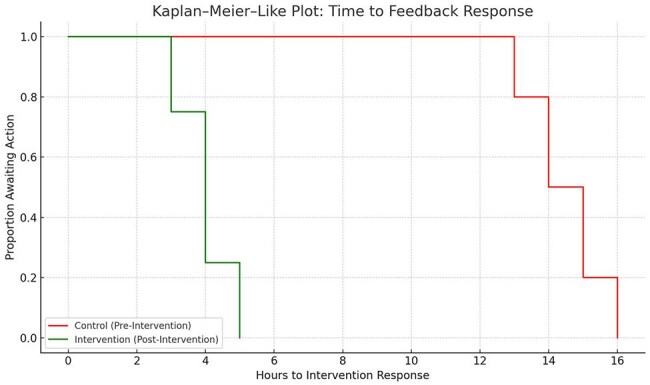
Kaplan–Meier–Like Plot: Time to Stewardship Intervention Response This step plot compares the timeliness of stewardship response to feedback before and after the mobile AMS platform implementation. The green line (post-intervention) shows a significantly faster decline, with median response time improving from 14.2 hours to 4.4 hours. The red line (pre-intervention) indicates delayed feedback action, highlighting the impact of digital workflow on timely AMS engagement.

**Methods:**

A prospective pre-post interventional study was conducted across two 18-month periods (pre: July 2021–Dec 2022; post: Jan 2023–June 2024). The mobile platform enabled daily antimicrobial audit, IV-to-oral switch tracking, escalation alerts, and prescriber feedback through digital workflows. Outcomes included inappropriate prescribing, de-escalation within 72 hours, guideline adherence, IV-to-oral conversion, intervention response time, and antibiotic consumption (DDD/1000 patient-days). Statistical analysis involved chi-square tests, logistic regression, and Kaplan–Meier survival analysis for intervention response.

**Results:**

A total of 4,643 prescriptions were audited (pre: 2,312; post: 2,331). Inappropriate prescribing declined from 30.7% to 18.2% (*p* < 0.001), while de-escalation within 72 hours improved from 26.1% to 55.8% (adjusted OR: 3.55, 95% CI: 2.84–4.45, *p* < 0.001). Guideline adherence rose from 59.8% to 83.9%, and IV-to-oral switch compliance increased from 21.7% to 50.1% (both *p* < 0.001). DDD/1000 patient-days for restricted antibiotics decreased by 28.6%. Recommendation acceptance improved from 48.5% to 82.3% (*p* < 0.001). Kaplan–Meier survival analysis demonstrated significantly faster response times to stewardship feedback post-intervention, with median response time dropping from 14.2 to 4.4 hours (*log-rank p* < 0.001).

**Conclusion:**

The mobile-based AMS platform significantly improved stewardship performance across key indicators. By digitizing surveillance, audit, and intervention workflows, the system enhanced accountability, clinical collaboration, and timeliness. These results support the platform’s value as a scalable, low-cost AMS solution for hospitals in low- and middle-income countries.

**Disclosures:**

All Authors: No reported disclosures

